# High glutathionylation of placental endothelial nitric oxide synthase in preeclampsia

**DOI:** 10.1016/j.redox.2019.101126

**Published:** 2019-01-26

**Authors:** Paul Guerby, Audrey Swiader, Nathalie Augé, Olivier Parant, Christophe Vayssière, Koji Uchida, Robert Salvayre, Anne Negre-Salvayre

**Affiliations:** a Inserm U-1048, Université de Toulouse, France; b Pôle de gynécologie obstétrique, Hôpital Paule-de-Viguier, CHU de Toulouse, France; c Laboratory of Food Chemistry, Graduate School of Agricultural and Life Sciences, University of Tokyo, Japan

**Keywords:** NO, nitric oxide, eNOS, endothelial nitric oxide synthase, iNOS, inducible nitric oxide synthase, O_2_^•–^, superoxide anion, ROS, reactive oxygen species, GSH, reduced glutathione, GSSG, oxidized glutathione, BH4, tetrahydrobiopterin, PE, preeclampsia, NO, ENOS, S-glutathionylation, Glutathione, Oxidative stress, O_2_, Pregnancy, Trophoblast, Migration, Preeclampsia

## Abstract

Decreased nitric oxide (NO) bioavailability plays a critical role in the pathophysiology of preeclampsia (PE). Recent evidence indicates that S-glutathionylation may occur on the endothelial nitric oxide synthase (eNOS), leading to eNOS uncoupling, characterized by a decreased NO production and an increased generation of superoxide anion (O_2_^•–^). We hypothesized that eNOS glutathionylation may occur in PE placentas and participate in eNOS dysfunction.

The glutathionylation of eNOS was investigated in thirteen PE-affected patients and in nine normal pregnancies. Immunofluorescence, confocal microscopy and western-blot experiments carried out on eNOS immunoprecipitates, revealed a high level of eNOS glutathionylation in PE placentas, mostly reversed by dithiotreitol (DTT), thus indicative of S-glutathionylation. In order to investigate whether eNOS glutathionylation may alter trophoblast migration, an important event occurring during early placentation, cultured HTR-8/SVneo human trophoblasts (HTR8) were exposed either to low pO_2_ (O_2_ 1%) or to pO_2_ changes (O_2_ 1–20%), in order to generate oxidative stress. Trophoblasts exposed to low pO_2_, did not undergo oxidative stress nor eNOS S-glutathionylation, and were able to generate NO and migrate in a wound closure model. In contrast, trophoblasts submitted to low/high pO_2_ changes, exhibited oxidative stress and a (DTT reversible) S-glutathionylation of eNOS, associated with reduced NO production and migration. The autonomous production of NO seemed necessary for the migratory potential of HTR8, as suggested by the inhibitory effect of eNOS silencing by small interfering RNAs, and the eNOS inhibitor L-NAME, in low pO_2_ conditions. Finally, the addition of the NO donor, NOC-18 (5 µM), restored in part the migration of HTR8, thereby emphasizing the role of NO in trophoblast homeostasis.

In conclusion, the high level of eNOS S-glutathionylation in PE placentas provides new insights in the mechanism of eNOS dysfunction in this disease.

## Highlights

• eNOS is S-glutathionylated in preeclamptic placentas.
• Oxidative stress evoked by pO_2_ changes, triggers eNOS S-glutathionylation in HTR8.
• pO_2_ changes alter the invasive potential of HTR8 in the wound closure model.
• The NO donor NOC-18 restores the invasive potential of HTR8.

## Graphical abstract

**Figure:**
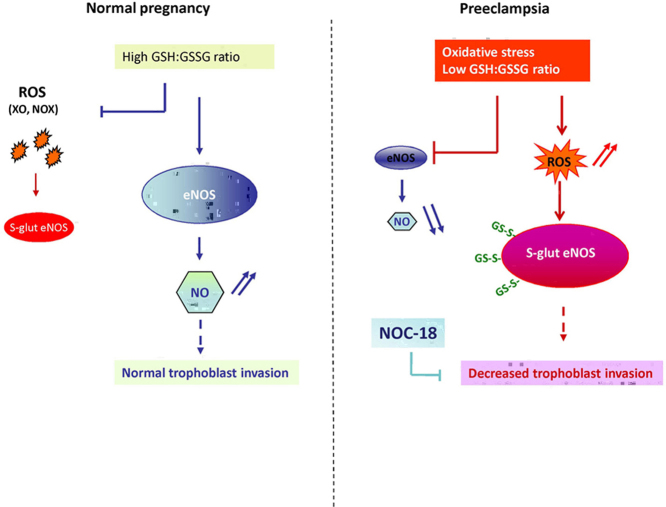

## 1 Introduction

Preeclampsia (PE), is a pregnancy-specific systemic vascular disorder that affects 3–7% of pregnancies in western countries and 4–18% in developing countries [1], [2]. It is classically diagnosed by hypertension and proteinuria observed after 20 weeks of gestation. If untreated, PE can lead to complications, including eclampsia, HELLP syndrome (elevated liver enzymes, haemolysis, and low platelets), pulmonary oedema, placental abruption. Moreover, PE is a cause of maternal and perinatal morbidity and mortality [2], [3]. Though the pathophysiology of PE has been deeply investigated and the clinical outcomes ameliorated, the underlying causes of poor placentation are not yet clarified [1].

It is generally admitted that in PE, the low resistance vasculature does not occur, due to abnormal cytotrophoblast invasion and defective spiral artery remodeling. The consequences are a decrease of blood flow to the placenta and an abnormal placentation due to abnormal hypoxia/oxygenation conditions in the intervillous space [2]. The hypoperfused placenta releases ‘placental antiangiogenic factors’ such as the soluble fms-like tyrosine kinase-1 (sFlt1) and s-endoglin into the maternal circulation [4], [5], [6], [7]. sFlt1 is a splice variant of VEGF receptor 1, and a soluble receptor of the proangiogenic vascular endothelial growth factor (VEGF) and placental growth factor (PIGF). In PE, there is an increased expression of sFlt1, concomitant with a decreased production of angiogenic PlGF and VEGF, leading to endothelial dysfunction and systemic inflammation [4], [5], [6], [7].

During normal pregnancy, important maternal circulatory changes (expanded plasma volume, increased cardiac output, peripheral vasodilation, increased vascular compliance) are associated with the adaptation of uteroplacental circulation, that is required for optimal placentation and embryo/fetal development [8], [9]. In early pregnancy, extravillous cytotrophoblasts invade and plug spiral arteries, thereby restricting blood flow, and leading to low pO_2_ (estimated < 20 mm Hg at 8–10 weeks) in the intervillous space. This relative hypoxia is required for cytotrophoblasts migration and to prevent oxidative stress in developing embryo that possesses only low antioxidant defenses at this stage of development [9], [10], [11].

Cytotrophoblasts participate in the remodeling of uterine spiral arteries that are transformed into large diameter and low resistance vessels with reduced pulsatility and weak vasoconstrictive ability. At 10–12 weeks, the trophoblastic arterial plugs dissolve, and uterine spiral arteries, remodeled into large diameter and low resistance vessels, increase progressively the oxygenated blood flow into intervillous space, thereby rising progressively placental O_2_ concentration (reaching about 60 mm Hg at 12–13 weeks) and inducing an extensive villous remodeling [8], [9], [10], [11].

In addition to histological changes of uterine arterial wall, the vasodilation of these arteries is mediated/reinforced by mediators, such as hormones (estrogens, progesterone), angiogenic growth factors (VEGF, PlGF) and vasodilator mediators such as nitric oxide (NO), prostacyclin, endothelium-derived hyperpolarizing factor (EDHF), hydrogen sulfide (H_2_S) [12], [13], [14]. In addition to vasodilation, NO is involved in uterine artery remodeling and placentation [12].

In PE, the deficiency of both cytotrophoblast invasion and remodeling of uterine spiral arteries may induce a maladaptation of uteroplacental circulation associated with intermittent maternal blood flow and hypoxia/reoxygenation events. This leads to oxidative stress and an imbalance of angiogenic/antiangiogenic factors (VEGF/PIGF *vs* sFlt1) that elicit placental cell stress and abnormal placentation, endothelial dysfunction and systemic inflammation [2], [4], [5], [6], [7], [10], [11].

Among the mechanisms involved in placenta dysfunction, the reduced bioavailability of NO and oxidative stress are thought to play a critical role in the maternal-placental circulation [12], [13], [14], [15], [16] and poor placentation [17], [18]. Moreover, the inhibition of nitric oxide synthase (eNOS) by L-NAME or genetic invalidation, is classically used for developing PE animal models [19]. A number of factors contribute to alter NO signaling, and are associated with an increased risk of PE, as recently summarized [20]. This includes alterations of eNOS regulation or function. For instance, eNOS polymorphism (G894T and T-786C) [21], [22], or eNOS uncoupling [17], [23], [24], have been associated with an increased risk of PE. A cause of eNOS uncoupling is the oxidation of its cofactor, (6 R)−5,6,7,8-tetrahydro-L-biopterin (BH4), which is highly sensitive to oxidative stress [25]. Other uncoupling mechanisms have been reported including an increased level of the endogenous NOS inhibitor ADMA (asymmetric dimethyl-l-arginine) [26], [27], or an increased arginase activity which reduces the availability of the eNOS substrate L-arginine [28].

A new mechanism of eNOS uncoupling, reported by Zweier's group [29], may result from its S-glutathionylation, a post-translational modification by oxidized glutathione of cysteine residues, specifically Cys689 and Cys908, that are critical to maintain eNOS function. The S-glutathionylation of cysteine residues of proteins is a reversible modification occurring under mild and severe oxidative stress conditions [30], [31], [32].

Since eNOS glutathionylation is a cause of reduced NO production, we investigated whether eNOS glutathionylation is increased in PE placentas, and whether such eNOS modification may occur in cultured trophoblast under oxidative stress conditions, and is associated with trophoblast dysfunction.

## 2 Methods

### 2.1 Materials

Anti-eNOS (ab5589) and anti-iNOS (ab3523) used for immunohistochemistry were from Abcam (Paris, France). Anti-eNOS antibody (AF950) used for immunoprecipitation experiments was from R&D Systems (Bio-Techne, France). Anti-glutathione antibody recognizing GS-S-proteins was from Virogen (Watertown, MA, USA). Secondary antibodies anti-mouse and anti-rabbit HRP-conjugated were from Cell Signaling Technology (Ozyme, France). Anti-Von Willebrand Factor (VWF) (AB7356) was from Chemicon (Merck Millipore) and anti-VEGF was from Sigma. Secondary anti-goat HRP-conjugated was purchased from Southern Biotech (Clinisciences, France). Secondary Alexa Fluor antibodies (488 and 546) were from Life Technologies (Courtaboeuf, France). Dihydroethidine (DHE), DAF-FM diacetate (4-amino-5-methylamino-2′,7′-difluorofluorescein diacetate), dithiotreitol (DTT), 4,6-Diamidino 2-phenylindole dihydrochloride (DAPI), oxypurinol, VAS2870, L-NAME (N-Nitro-L-arginine methyl ester hydrochloride), BH4 (tetrahydrobiopterin dihydrochloride) were from Sigma-Aldrich (Saint Quentin Fallavier, France). 2′,7′-Dichlorodihydrofluorescein diacetate (H_2_DCFDA) and SYTO-13 were from Thermofisher (Villebon sur Yvette, France), NOC-18 (diethylenetriamine/nitric oxide adduct; DETA NONOate), was from Santa Cruz Biotechnology (Clinisciences, France).

### 2.2 **Placental tissue collection**

The use and study of human placentas were approved by the Research Ethic Committee of Toulouse University Hospital (CER number 03–0115). Two groups of age-matched pregnant women were analyzed, one normotensive control group established from uncomplicated pregnancies (n = 9, mean gestational age 39 weeks)**,** and one group exhibiting severe PE features (n = 13, mean gestational age 29 weeks). The clinical details are summarized in Table I. Placentas from normal and PE pregnancies were recovered from elective cesarean section (University Hospital Center of Toulouse, France).

**Table I:** Placental tissue collection.

**Patient characteristics**	***Normal***	***Preeclampsia***	***p-value***
	***(n = 9)***	***(n = 13)***	
**Maternal age, mean (SD)**	30.5 (4.1)	31.1 (6)	0.82
**BMI**	26.7 (3.7)	24.2 (4)	0.17
**Parity**	1.8 (1.0)	1.4 (0.7)	0.32
**Blood sampling**			
**Systolic blood pressure (mmHg), mean (SD)**	118.9 (8.2)	159.6 (11)	< 0.001
**Diastolic blood pressure (mmHg), mean (SD)**	70.5 (5.8)	97.5 (9.4)	< 0.001
**Proteinuria (g/24 h) mean, (SD)**	<0.3	5.4 (5)	< 0.01
**Delivery**			
**Gestational age (wk), median (IQR)**	39 (1)	29 (3.2)	< 0.001
**Birth weight (g), mean, (SD)**	3383.9 (479.0)	1108.3 (423.3)	< 0.001

P value: P < 0.05 statistically significant; BMI: body mass index; results are expressed as means ± SD or medians ± IQR.

Preeclampsia was defined according to the American College of Obstetricians and Gynecologists, i.e. by a systolic blood pressure (SBP) greater than or equal to 140 mm Hg, or a diastolic blood pressure (DBP) greater than or equal to 90 mm Hg and a proteinuria greater than or equal to 300 mg per 24-h urine collection, after 20 weeks of gestation. Severe PE features included fetal and maternal complications (pulmonary oedema, myocardial infarction, stroke, acute respiratory distress syndrome, coagulopathy, severe renal failure, and retinal injury) leading to delivery induction before 34 weeks of gestation. For each patient, the placenta was immediately collected after delivery, and washed four times in ice-cold PBS to remove remaining blood. Some fragments were fixed into 4% paraformaldehyde and embedded in paraffin for immunofluorescence analysis. Other fragments were kept frozen at − 80 °C until use. As illustrated in supp. Fig. I, histological analysis (hemalun/eosin) of PE placentas, showed characteristic features of increased syncytial knots, distal villous hypoplasia and calcification deposits.

### 2.3 Cell culture

HTR-8/SVneo cells (HTR8) were a generous gift from Dr Charles H. Graham (Queen's University, Kingston, ON, Canada) [33]. This cell line was established from explant culture of first-trimester human placenta and immortalized by the simian virus 40 large T antigen. Under standard conditions, HTR8 were cultured in RPMI supplemented with 5% fetal bovine serum (FBS) (5% CO_2_, 20% O_2_, 37 °C). For the experiments, subconfluent HTR8 were incubated in FBS-free RPMI and put in a hypoxic chamber (37 °C, 5% CO_2_, 1% O_2_). Exposure to low (1% O_2_) *vs* high (20% O_2_) pO_2_ experiments for inducing oxidative stress, were carried out as reported in [34], modified as follows: cells were maintained in low pO_2_ (O_2_ 1%) for 18 h, then exposed to high pO_2_ (O_2_ 20%) for 2 h and re-exposed to 1% O_2_ for 4 h (or as indicated in legends). Before exposure to high pO_2_, the hypoxic medium was removed and replaced by fresh RPMI medium. Cell viability was estimated using the MTT [3-(4,5dimethylthiazol-2-yl)− 2,5-diphenyltetrazolium bromide] assay [35].

siRNA transfections were carried out using ON-TARGET plus human NOS3 siRNA (006490, Dharmacon) with the Hiperfect reagent (301705, Qiagen) according to manufacturer's instructions.

### 2.4 Intracellular ROS, O_2_^•–^ and NO determination

Intracellular O_2_^•–^ generation was detected using the dihydroethidine (DHE) probe. Live cells were stained with DHE (5 µM) and counterstained with the permeant DNA probe Syto 13 (0.5 µM), for 15 min at 37 °C, and the fluorescence was monitored by microscopy, in the conditions reported by Chen et al. [29], or was measured in PBS (Spectrofluorometer Hitachi F-2500, exc/em 530/610 nm) [36], [37].

Alternatively, ROS were evaluated using the oxidation of the non specific H_2_DCFDA-AM probe [37]. The probe was added to the culture medium (5 µM final concentration) 30 min before the end of the experiment. After washing in PBS, the fluorescence was measured in PBS (exc/em 495/525 nm). The data are expressed as ratio of the low pO_2_ control.

NO production was determined in HTR8 preincubated for 30 min using the DAF-FM diacetate probe (5 µM). After washing with PBS, NO production was monitored fluorometrically (exc/em 495/525 nm). To ensure the specificity of NO production which may vary using DAF-FM probe [37], a negative control was done in the presence of L-NAME (100 µM), and substracted to each measurement. NO production was expressed as % of the low pO_2_ control/h.

### 2.5 Wound closure assay for evaluation of the HTR8 invasive potential

HTR8 were cultured up to confluency on sterile coverslips in 30 mm sterile dishes. A scratch was made with a sterile 200 µl plastic pipette tip, and cells were put in low pO_2_ conditions for 48 h (control) or were submitted to pO_2_ changes, as above described. Alternatively, the effect of eNOS inhibition on HTR8 migration, was studied using L-NAME (100 µM) or eNOS silencing by siRNAs, in HTR8 maintained in low pO_2_ (O_2_ 1%) for 48 h. At the end, cells were washed with PBS, fixed with 4% paraformaldehyde, and permeabilized for 5 min with 0.25% Triton X-100 in PBS. After DAPI staining, the coverslips were mounted on a glass slide and analyzed by fluorescence microscopy.

### 2.6 Immunofluorescence studies

Serial 3 µm thin sections of placentas were dewaxed, rehydrated and used for immunofluorescence analysis. The glutathionylation of eNOS was analyzed under conditions described by Chen et al. [29], using mouse anti-glutathione recognizing GSS-proteins and rabbit anti-human eNOS primary antibodies, followed by secondary anti-mouse Alexa fluor-488 and anti-rabbit Alexa fluor-546 conjugated antibodies. Alternatively, dewaxed and rehydrated slides were treated by DTT (1 mM) for 30 min at room temperature, washed in PBS, and immunofluorescence studies were carried out. Nuclei were stained with DAPI (1 µg/mL). The slides were analyzed by confocal microscopy (Zeiss LSM780). The percentage of eNOS overlapping with GSS-protein was determined using the plug-in JACoP of ImageJ software, by evaluating the Mander's coefficient.

### 2.7 Preparation of placental homogenates

Placental tissues (20 mg) were placed into a homogenate plastic tube containing 1 mL of ice-cold PBS and protease inhibitors. The tissue was homogenized with a PreCellys homogenizer for twice 30 s on ice. Homogenates were centrifuged at 13,000 rpm for 10 min at 4 °C. The supernatant was collected, aliquoted and stored at − 80 °C until required.

### 2.8 Reduced vs oxidized glutathione content

The ratio of reduced (GSH) to oxidized glutathione (GSSG) was determined in control and PE homogenates using the GSH/GSSG Ratio Detection Assay Kit (Abcam, Paris, France).

### 2.9 Immunoprecipitation and western-blot experiments

Control and PE placenta homogenates, or HTR8 cell extracts were used for eNOS immunoprecipitation. After solubilization in lysis buffer (50 mM Tris-HCl pH7,4, 150 mM NaCl, Igepal 1%, protease and phosphatase inhibitors), protein extracts (1 mg) were incubated overnight at 4 °C with a human anti-eNOS antibody followed by precipitation for 2 h at 4 °C with protein-A sepharose coated beads. The beads were washed three times with PBS, resuspended with loading buffer in non-reducing or reducing conditions with or without DTT ( 1 mM DTT for 30 min at room temperature) and boiled for 5 min. The protein complexes were subjected to Western-blot analysis. The protein extracts were separated by 7% SDS-PAGE and transferred to PVDF membrane (Millipore). The membranes were blocked with 5% nonfat milk/TBST for 2 h and incubated with glutathione or eNOS antibodies (1:1000) overnight at 4 °C. After washing, the membranes were incubated with horseradish-peroxidase-conjugated secondary antibodies (1:5000) for 1 h.

### 2.10 Statistical analysis

The results are expressed as mean ± SEM. For normally distributed data, Student's *t*-test was used, otherwise nonparametric Mann-Whitney *U*-test was employed. Statistical calculations were carried out using the software Graphpad Prism, version 6.01 (Graph Pad Software Inc., CA, USA). Values of *p* < 0.05 were considered significant.

## 3 Results

### 3.1 S-Glutathionylation of eNOS in human placentas

**Fig. 1:**
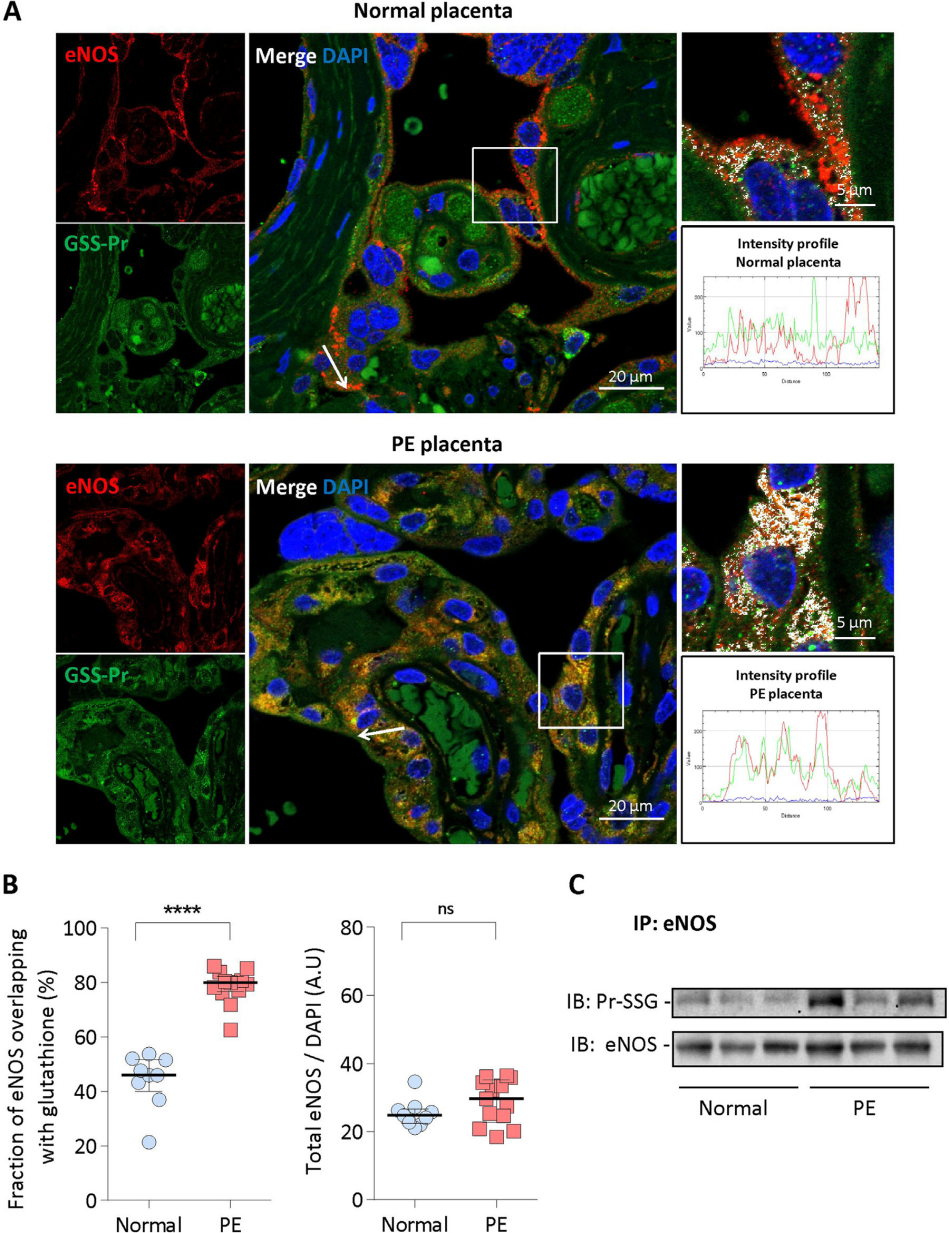
**Glutathionylation of eNOS in control and PE placentas.** A, Representative pictures of eNOS glutathionylation showing the colocalization of eNOS (red) and glutathione (green) in placentas from one normal pregnancy (upper picture) and one PE patient (lower picture) (scale bar, 20 µm). Co-immunofluorescence staining and confocal microscopy were carried out on placenta sections using anti-polyclonal eNOS antibody and anti-glutathione antibody recognizing GSS-proteins. Nuclei were counterstained with DAPI. Yellow area show the colocalization of eNOS with glutathione on the composite image (merge). Inserts indicate area selected for higher magnification (colocalization of eNOS and glutathione in white areas, scale bar 5 µm). The fluorescence intensity profiles indicate the colocalization of eNOS (red) and GSH (green) along the arrow, in PE *vs* normal placentas. These pictures are representative of all studied normal and PE placentas (and see also Supp Fig. II). B. Quantification from confocal pictures, of glutathione-modified *vs* total eNOS (left) and total eNOS *vs* DAPI (right), in normal (n = 9) and in PE placentas (n = 13). Total eNOS was quantified by the ratio between red-fluorescence and the number of DAPI-stained nuclei. The quantification of eNOS fluorescence overlapping with GSH fluorescence, was based on Mander's coefficient analysis. Data represent median±interquartile range. C, Immunoprecipitation and western-blot experiments showing eNOS glutathionylation in placentas from normal (3) and PE (3) pregnancies. Statistical significance was assessed using the nonparametric Mann-Whitney *U* test (ns, non significant; ***, *p* < 0.001).

Immunofluorescence staining with anti-glutathione recognizing GSS-protein and anti-eNOS antibodies showed an apparent colocalization between eNOS (red) and GS-S-protein (green) in PE and at a lesser extent in normal placentas (Fig. 1A and Supp. Fig. II). The quantification of eNOS glutathionylation was evaluated by measuring the ratio of glutathionylated eNOS (white) *vs* total eNOS (red). In placentas from normal pregnancies, the ratio glutathionylated/total eNOS was around 45%, whereas it reached more than 80% in PE placentas (Fig. 1B). Total eNOS protein expression, evaluated by the ratio eNOS/DAPI, was similar or slightly higher in PE compared to normal placentas (Fig. 1B). eNOS was highly expressed in syncytiotrophoblasts and in endothelial cells of microvessels, where it colocalized with the von Willebrand factor, a specific endothelial cell marker (Supp Fig. III). As inflammation is a classical component of PE [9], [15], [16], [17], [18], we also investigated the expression of the inducible nitric oxide synthase (iNOS), which was comparable in PE and normal placentas (Supp. Fig. IV), in agreement with previous reports [38]. It is to note that iNOS was much less glutathionylated than eNOS, and no differences in iNOS glutathionylation expression were observed between normal and PE placentas (Supp. Fig. IV).

Western-blot experiments were carried out on eNOS immunoprecipitates prepared from placenta homogenates, and confirmed the lack of difference in total eNOS expression, and the high eNOS glutathionylation levels in PE when compared to normal placentas (Fig. 1C).

**Fig. 2:**
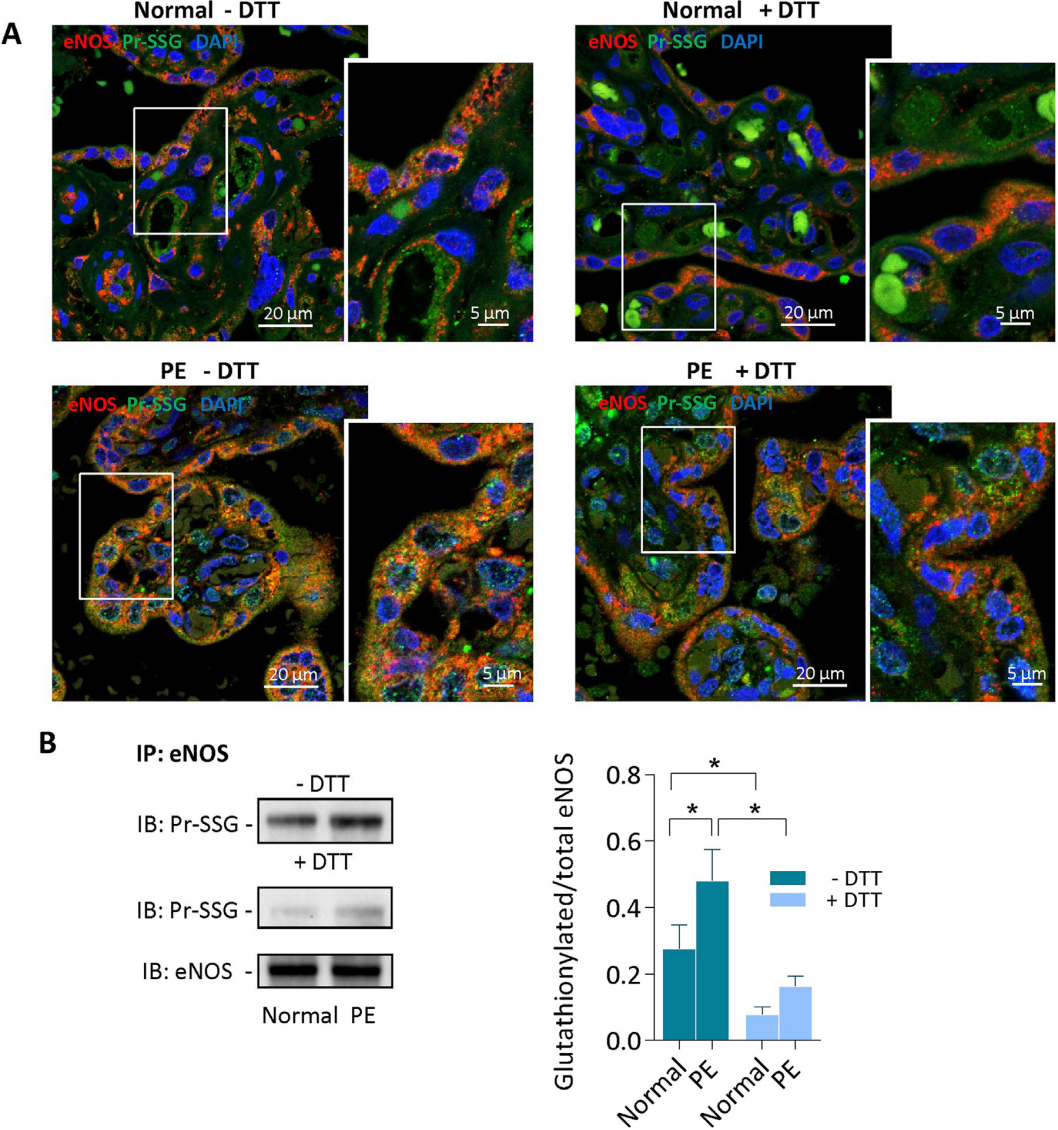
**Reversion of eNOS glutathionylation by DTT in placentas.** A, Representative pictures of DTT-sensitive eNOS glutathionylation in placentas from one normal pregnancy (upper panel), and one PE patient (lower panel). Slides were preincubated with DTT (1 mM, 30 min room temperature), before immunofluorescence staining and confocal microscopy, as indicated in Fig. 1 (scale bar 20 µm). In insert, higher magnification picture (scale bar 5 µm). B, Immunoprecipitation and western-blot experiments showing the reversible eNOS glutathionylation in placentas, after DTT treatment of the protein extract. Left, representative western-blot picture; Right, expression of the data as median with interquartile range. The statistical analysis was assessed using a Mann-Whitney test. *p < 0.05.

To investigate whether eNOS is S-glutathionylated, i.e. sensitive to reducing agents, placenta slides were mildly treated by dithiotreitol (DTT) (1 mM, 30 min), before immunostaining with anti-glutathione and anti-eNOS antibodies. As shown in Fig. 2A, most of eNOS glutathionylation was reversed by DTT in normal and PE placentas, indicating that placental eNOS was mainly S-glutathionylated. It is to note that a minor fraction of glutathionylated eNOS was resistant to DTT treatment of immunoprecipitated samples (1 mM, 30 min) in PE placentas (Fig. 2B), suggesting that the major part of placental glutathionylated eNOS undergoes (DTT-reversible) S-glutathionylation and a minor part could be (DTT-resistant) C-glutathionylated [39].

As the ratio of GSH to glutathione disulfide (GSSG) regulates the intracellular redox status, and its imbalance may lead to protein S-glutathionylation [30], the GSH and GSSG levels were evaluated in placenta homogenates. Data in supp Fig.V, point out a significant decrease of the GSH/GSSG ratio in PE placentas, indicative of oxidative stress and redox status imbalance.

### 3.2 Human trophoblasts HTR8 exposed to pO_2_ variations exhibit eNOS S-glutathionylation

As eNOS is expressed in trophoblasts and is involved in placentation and trophoblast migration into spiral arteries [12], and as oxidative stress is thought to disturb placentation and remodeling of uterine spiral circulatory by extravillous trophoblasts [9], we used a trophoblastic cell line to investigate whether oxidative stress may induce eNOS glutathionylation and subsequent cellular dysfunction. For this purpose, we used the HTR-8/SVneo cytotrophoblast cell line (HTR8), which was established by Graham et al. [33] from first trimester human trophoblasts. These immortalized cells exhibit a stable phenotype during cell culture, and express eNOS.

HTR8 in FBS-free culture medium, were exposed to low pO_2_ (1% O_2_), in which they migrate and proliferate [40]. Oxidative stress was generated by pO_2_ changes (1–20%). These conditions did not elicit any loss of HTR8 viability (Supp. Fig.VI). Low pO_2_ stimulated the expression and stabilization of the hypoxia-sensitive transcription factor Hif1α [41], [42], in agreement with previous studies in HTR8 [43]. In contrast, Hif1α was unstable in cells exposed to high pO_2_ (20% O_2_), or submitted to pO_2_ changes [41] (Supp. Fig.VII).

**Fig. 3:**
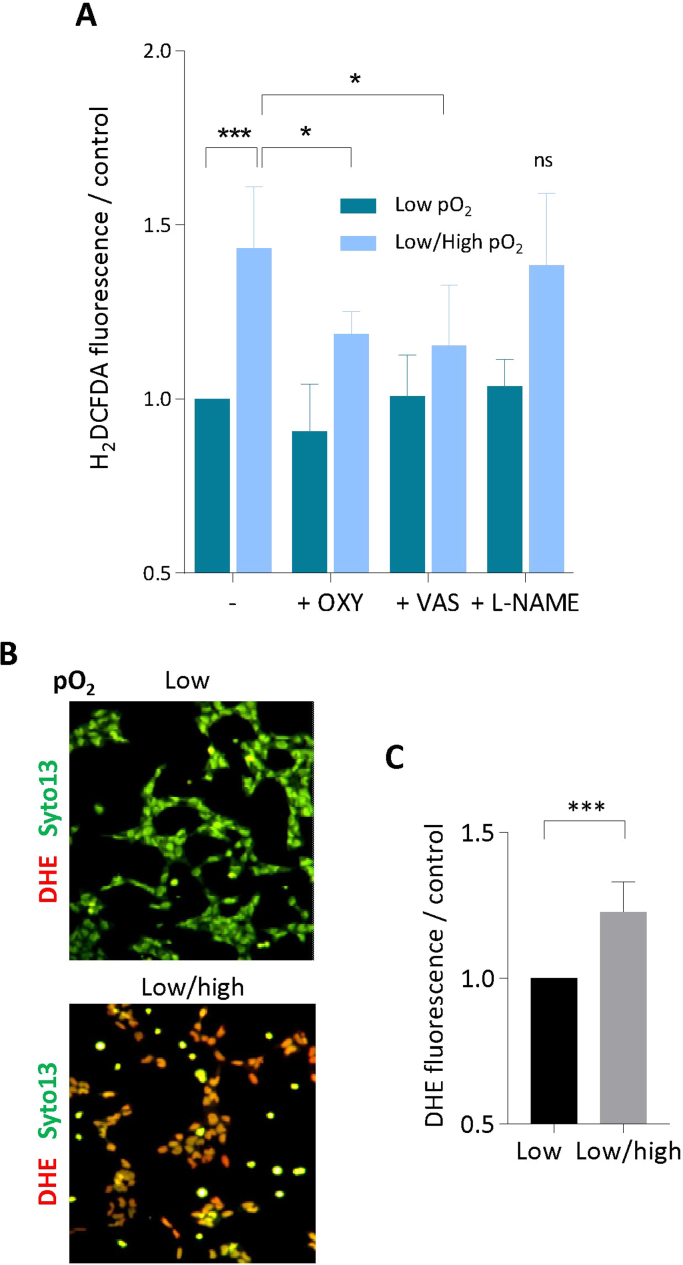
**Intracellular ROS increase and O**^**•−**^**generation evoked by pO**_**2**_**changes in HTR8.** A, Intracellular ROS detected using the H_2_DCFDA-AM fluorescent probe, in HTR8 maintained in low pO_2_ (O_2_ 1%) or submitted to pO_2_ changes, and effect of inhibitors L-NAME (100 µM), oxypurinol (OXY) (1 mM) and Vas2870 (VAS, 5 µM). H_2_DCFDA-AM was added to the HTR8 culture medium (5 µM) 30 min before the end of incubation. Cells were washed in PBS, lyzed in water, and the fluorescence was measured (exc. 495 nm/em. 525 nm). B, C, Detection of intracellular O_2_^•−^ using the dihydroethidine probe (DHE). B, representative pictures of HTR8 showing the production of O_2_^•−^ in low pO_2_ or exposed to pO_2_ changes. DHE (5 µM) and the cell permeant Syto13 (for nuclei counterstaining), were added to the culture medium at the end of incubation, and the fluorescence of cells was immediately monitored. C, Measurement of O_2_^•−^ production by HTR8. DHE fluorescence was measured in cell homogenates in PBS (Spectrofluoremeter Hitachi 2500, exc. 530 nm/em. 610 nm). Results are expressed as ratio of the low pO_2_ control. Means ± SEM of 4 separate experiments, statistical analysis with a Student *t*-test. * **p < 0.001.

The exposure of HTR8 to pO_2_ changes stimulated the production of intracellular ROS, detected using the nonspecific DCF-DA probe [37], which were partly inhibited by Vas2870, a pan NADPH oxidase inhibitor, and by the xanthine oxidase inhibitor oxypurinol (Fig. 3A), indicating a role for xanthine oxidase, in agreement with [34]. ROS production included the generation of O_2_^•–^, detected using dihydroethidine (DHE, a cell-permeant O_2_^•–^ sensitive probe ) [36] (Fig. 3B,C).

**Fig. 4:**
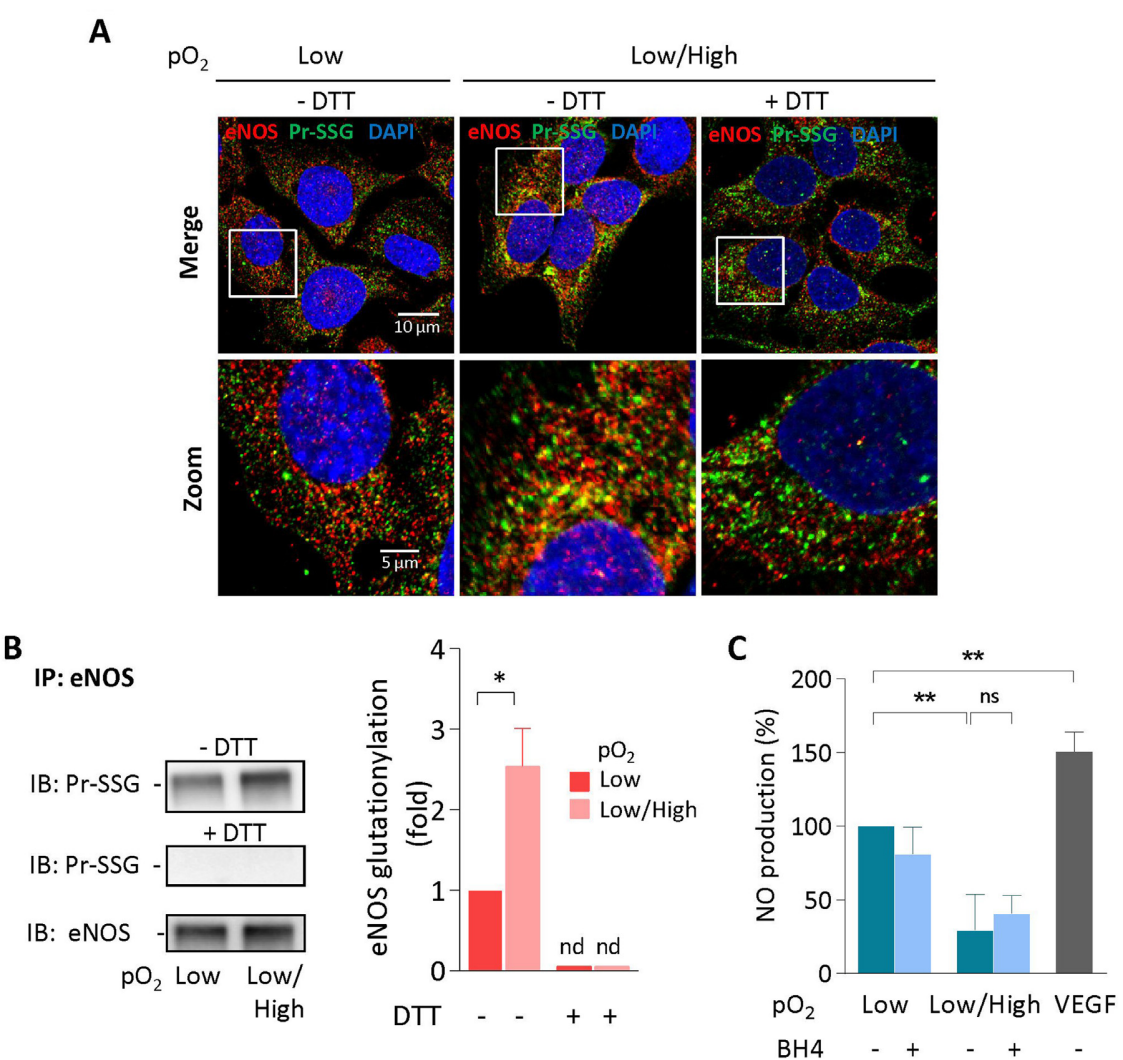
**pO**_**2**_**changes elicit eNOS S-glutathionylation and dysfunction in HTR8.** A, Representative pictures of eNOS and glutathione expression in HTR8 incubated in low pO_2_ (O_2_ 1%) (left panel), or pO_2_ changes (18 h O_2_ 1%-2 h O_2_ 20%-2 h O_2_ 1%) before (middle panel), and after (right panel) DTT treatment (1 mM, 30 min, room temperature). The colocalization of eNOS and glutathione was studied as described in the legend to Fig. 2 and in the Method Section. Upper panels, scale-bar 10 µm, lower panels, scale-bar 5 µm. B, Immunoprecipitation and immunoblotting experiments showing the glutathionylation of eNOS in HTR8 submitted to low pO_2_ or pO_2_ changes. The reversibility of glutathionylation was studied by preincubating eNOS immunoprecipitates with DTT (1 mM, 30 min, room temperature), before immunoblotting. C, Measurement of NO production by HTR8 in low pO_2_ conditions or exposed to pO_2_ changes. DAF-FM diacetate probe (5 µM) was added to the HTR8 culture medium (5 µM) 30 min before the end of the incubation. The production of NO was fluorometrically recorded (exc/em 495/525 nm) for 60 min in the presence or absence of BH4 (10 µM). A negative control using L-NAME (100 µM) was done concomitantly, and the L-NAME-insensitive residual fluorescence was substracted to each point, for eliminating the non NO-specific DAF fluorescence. A positive control was done by stimulating HTR8 with VEGF (20 nM, 15 min). The results are expressed as % of NO produced by the control in low pO_2_, means ± SEM of 4 separate experiments, statistical analysis with a Student *t*-test. *p < 0.05; ns, non significant.

In HTR8 exposed to pO_2_ changes (i.e. oxidative stress), immunofluorescence and confocal microscopy pointed out the glutathionylation of eNOS (i.e. its colocalisation with glutathione) (Fig. 4A), which was confirmed by immunoprecipitation and western blot experiments (Fig. 4B). The DTT treatment suppressed the overlay of immunofluorescence of GS-S-protein and eNOS as well as the glutathionylation of eNOS on western blot (Fig. 4A,B), indicating that eNOS is S-glutathionylated in HTR8 submitted to pO_2_ changes.

The production of NO was quantified using the fluorescent NO-sensitive probe DAF-FM. The generation of NO by cells exposed to pO_2_ changes, was significantly decreased, when compared to cells maintained in 1% O_2_ (Fig. 4C). The addition of BH4 (10 µM) did not restore the production of NO, indicating that the decrease of eNOS activity induced by oxidative stress, could not result from BH4 oxidation, and that BH4 cannot compensate when eNOS is inactive.

### 3.3 HTR8 migration is reduced under conditions of eNOS S-glutathionylation and is partly restored by NO donor

NO plays an important role throughout pregnancy, and particularly in the early steps of placentation, where it contributes to the invasion of spiral arteries by cytotrophoblasts [8], [9], [10], [11], [17], [18]. To test the role of NO in trophoblast migration and the potential pathogenic role of eNOS glutathionylation induced by oxidative stress, we used the ability of HTR8 to migrate in the wound closure assay [40].

**Fig. 5:**
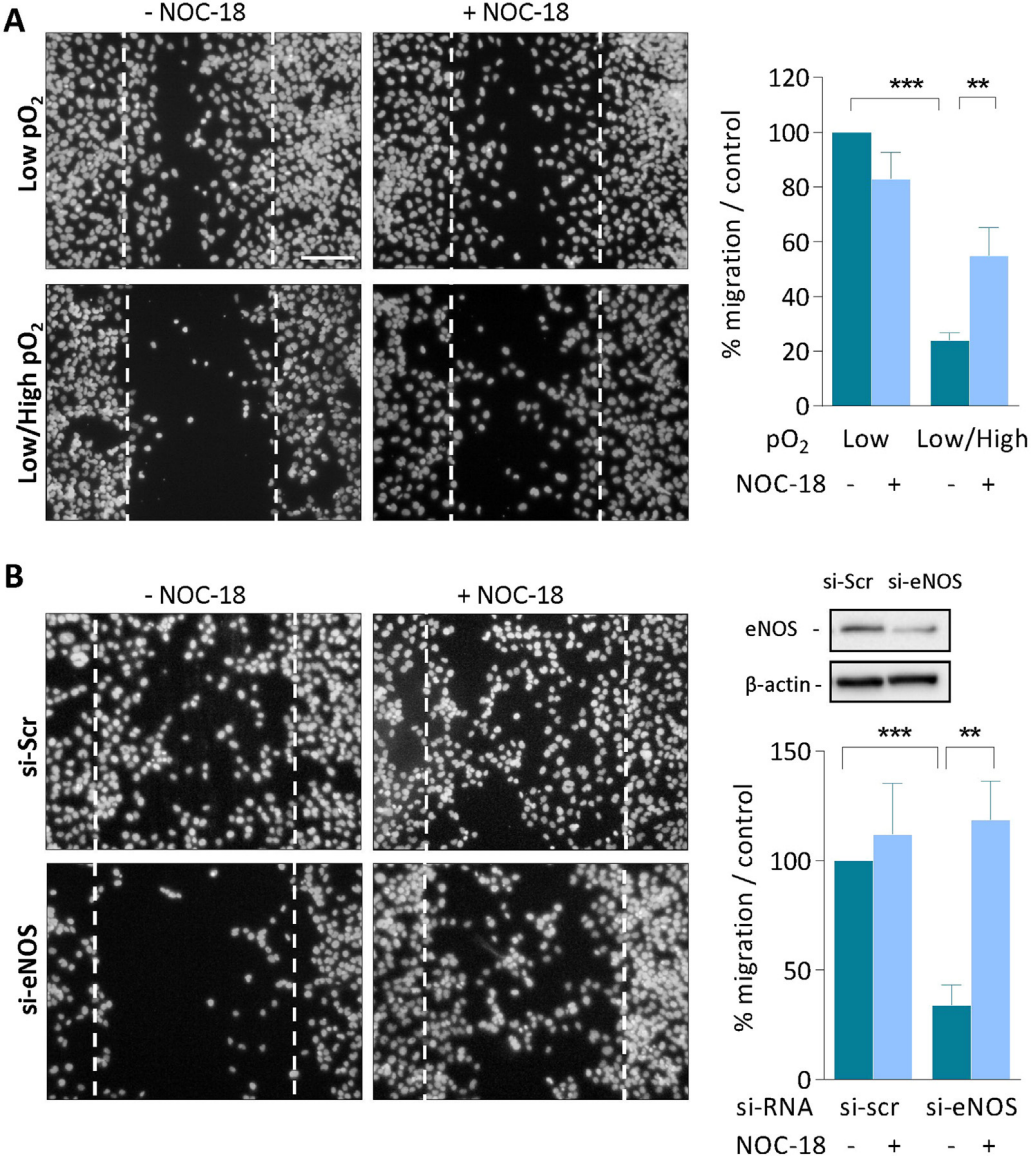
**NO is required for HTR8 migration in the wound closure assay.** A scratch was made on confluent HTR8, before incubation in low pO_2_ or pO_2_ change conditions (A), as in Fig. 4. Alternatively, HTR8 were maintained in low pO_2_ for 48 h in the presence of siRNAs specifically targeting NOS3 (eNOS) (B). A control was done using a scrambled si-RNA mixture (Scr). The efficacy of siRNAs was evaluated on eNOS expression by western-blot, using β-actin as control (upper panel). The effect of NOC-18 (5 µM) was tested on HTR8 in each condition. Scale bar= 100 µm. A negative control was done with a wound scratch extemporaneously made on HTR8 at the end of the experiment. The quantification of HTR8 invasive capacity was evaluated by counting the cells having migrated within the wound in each condition. Means±SEM of 4 separate experiments, statistical analysis with a Student *t*-test. * *p < 0.01; * **p < 0.001.

As shown in Fig. 5, HTR8 grown in serum-free medium, at low pO_2_ (O_2_ 1%), migrated within the wound through a cell-autonomous NO-dependent mechanism, as suggested by the inhibition of HTR8 migration induced by the silencing of eNOS by siRNAs (Fig. 5), or by the eNOS inhibitor L-NAME (Supp.Fig.VIII). Interestingly, under oxidative stress conditions (exposure to pO2 changes) inducing eNOS glutathionylation, HTR8 migration was inhibited, but was compensated (in part) by the NO donor NOC-18, which also restored the migration of cells either eNOS-silenced by siRNAs (Fig. 5). These data support the hypothesis that cell autonomous production of NO by eNOS is required for trophoblast migration during the early steps of placentation and remodeling of uterine spiral arteries, and that oxidative stress induced by pO_2_ changes induces S-glutathionylation and inhibition of eNOS, resulting in deficient cytotrophoblast migration. It is to note that a constant exposure to high pO_2_ (O_2_ 20%) in serum-free medium, did not stimulate HTR8 migration within the wound (data not shown).

## 4 Discussion

The decreased NO bioavailability in the first trimester of pregnancy, is a cause of poor placentation [8], [9], [10], [11], [13], while eNOS deficiency is associated with reduced placental vascularization and fetal growth restriction in homozygous eNOS^-/-^ conceptus, but not in heterozygous [44]. In PE, the molecular basis of eNOS dysfunction are still unclear. In this study, we report for the first time that eNOS is highly S-glutathionylated in placentas from PE patients. As the reversible (reductant-sensitive) S-glutathionylation leads to eNOS uncoupling and a decrease in NO production [29], the results reported here may partly explain the molecular and pathophysiological mechanisms leading to eNOS dysfunction and reduced NO generation in PE.

A first point is that eNOS was found glutathionylated in all placentas, with levels reaching around 45% of total eNOS in placentas from normal pregnancy, *vs* more than 80% in PE placentas. No major differences were observed concerning the total eNOS expression in placentas, in agreement with previous studies [38], [45], and in contrast to the decrease in eNOS expression reported by Du et al. [46]. These discrepancies could be due to the severity of PE cases (higher in [38], [45] and in our study), which could be associated with an increased eNOS expression [46]. Most part of eNOS glutathionylation was reversed by the reducing agent DTT in normal and PE placentas, indicating that eNOS is mainly S-glutathionylated, i.e. undergoes a post-translational reversible modification of cysteine residues by oxidized glutathione, according to the mechanism described by Chen et al. [29]. This high level of eNOS S-glutathionylation, with less than 20% of non-glutathionylated, thus still active eNOS, may constitute a mechanism of decreased NO bioavailability, with consequences on placentation [9], [10], [11], uterine artery contractility, placental circulation and maternal blood pressure regulation [17], [18].

S-glutathionylation is a post-translational modification of proteins occurring as an adaptative response to oxidative stress and loss of intracellular reductive ability, and a mechanism protecting oxidant-sensitive thiol of cysteine residues from irreversible modifications [29], [30], [31]. The S-glutathionylation of eNOS was reported in the vascular wall of hypertensive rats [29], and in endothelial cells exposed to hypoxia/reoxygenation [34], or mutant for glutamate-cysteine ligase, in which the biosynthesis of glutathione is altered [47], but to our knowledge, this is the first report showing that eNOS is S-glutathionylated in placentas.

In early pregnancy, extravillous cytotrophoblasts invade and plug the maternal uterine spiral arteries, thereby decreasing blood flow and lowering pO_2_. This relative hypoxia, necessary for promoting cytotrophoblast migration, and spiral artery remodeling [9], [10], [11], maintains a low local production of ROS and prevents oxidative stress that would be highly deleterious because of the low expression of antioxidant systems in the early stage of placenta and embryo development [9], [10], [11], [48]. In the same way, a local NO production is required for optimal trophoblast migration and uterine spiral artery remodeling [12], [44]. In our HTR8 experimental model system, a relative hypoxia is associated with a moderate eNOS glutathionylation and NO production sufficient for HTR8 migration.

Later on, during normal pregnancy, the removal of trophoblastic plugs and the remodeling of placental vessels, allow progressively increasing the blood flow into placental intervillous spaces, while the expression of antioxidant enzymes increases in the placenta and embryo [9]. A low to moderate oxidative stress apparently occurs throughout normal pregnancy [9], that may explain the moderate eNOS glutathionylation observed in normal placentas at the end of the pregnancy. It may be noted that this moderate eNOS glutathionylation (estimated to around 45%) allows generating around 70% of the maximal rate of NO production (60% by non-modified eNOS and 10% by S-glutathionylated eNOS, since highly S-glutathionylated eNOS retains approximately 30% of its activity) [29]. This rate of NO production (70%) is compatible with a normal placentation, the remodeling of uterine spiral arteries and the embryo development, like that observed in heterozygous eNOS^+/-^ embryos [44].

In contrast, oxidative stress conditions (generated by pO_2_ changes), increase eNOS glutathionylation, and reduce NO production and trophoblast migration, as observed in the HTR8 experimental model system. This is in agreement with the hypothesis of Burton and Jauniaux stating that in the early stages of placenta development, excessive blood flow in the intervillous space may induce an oxidative stress that impairs trophoblast migration, vascular remodeling and placentation [10], [11], [12], [44], [48].

Later on, during PE pregnancy, the defect of remodeling of uterine spiral arteries maintains an intermittent contractility, leading to unsteady placental blood flow that generates local oxidative stress conditions. The persistent oxidative stress may explain the high level of eNOS glutathionylation in PE placentas. As highly glutathionylated eNOS is uncoupled, with loss of NO generation and gain of O_2_^•–^ production [29], this may participate to impair uterine spiral artery remodeling and enhance a local oxidative stress, possibly worsened by intermittent episodes of ischemia/reperfusion, and finally leading to a reduction of the placental blood flow.

In placentas, ROS could be produced by various sources, including xanthine oxidase, mitochondrial respiratory chain, or NOXs [9], [48], [49]. In PE, oxidative stress can be worsened by risk factors (preexisting hypertension, diabetes, obesity or disturbed immune response…), which are classically characterized by high rates of ROS and oxidative damages [15], [16], [48], [49]. These high oxidative stress conditions are known to cause an excessive and persistent S-glutathionylation of eNOS and other targets in diseases such as diabetes, renal fibrosis, Alzheimer, cataract, and cancer [30], [31], [32], [47], [50].

S-glutathionylation may modify the protein function and compromise the cell fate, when occurring on critical cysteine residues [30], [31]. The S-glutathionylation of eNOS occurs on Cys 689 and Cys 908, which are critical to maintain the normal function of the enzyme [29]. Consequently, S-glutathionylation triggers eNOS uncoupling, switching the protein activity from a NO producing enzyme towards a NADPH oxidase activity producing O_2_^•–^, with consequences on the pathophysiology of cardiovascular and renal diseases [29], [30], [31], [32], [49], [50]. A consequence is that S-glutathionylation of eNOS may per se maintain or aggravate ROS production and oxidative stress. Another major consequence is the decreased NO production due to eNOS dysfunction. An important function for NO, as for growth factors and H_2_S, is to promote the migration of cytotrophoblasts towards spiral arteries in the first weeks of pregnancy [17], [18]. In our model, this role of NO was demonstrated by the lack of HTR8 migration evoked by L-NAME, or by the silencing for eNOS by siRNAs. Likewise, pO_2_ changes leading to eNOS S-glutathionylation, inhibited the migration of HTR8. The key-role of NO on trophoblast migration, was emphasized by the benefit exerted by the NO donor, NOC-18 [51], which significantly restored cell migration in HTR8 silenced for eNOS or submitted to pO_2_ changes. So far, several NO donors have been tested on the late events of PE, in particular transdermal glyceryl trinitrate or transdermal isosorbide dinitrate patches, which improved the maternal blood pressure and fetoplacental circulation [52], [53]. Likewise, S-nitrosoglutathione (GSNO), was used for the treatment of HELLP syndrome and severe PE, with an improvement of blood pressure and platelet activation [54]. The protective effect of NOC-18 on HTR8 migration suggests that this NO donor could improve early events such as the invasive potential of trophoblasts and placentation, thereby allowing to prevent the development of PE.

## 5 Conclusion

In conclusion, our data highlight a high level of eNOS S-glutathionylation in preeclamptic placentas, which may possibly trigger eNOS dysfunction and decrease NO bioavailability throughout pregnancy. The benefit exerted by NOC-18, confirms the potential interest of NO donors for compensating the lack of NO and preventing the pathological process of PE.
