# Supplementary material for: High glutathionylation of placental endothelial nitric oxide synthase in preeclampsia
Source: Redox Biol. 2019 Jan 26;22:101126. doi: 10.1016/j.redox.2019.101126 (PMC6370867; doi:10.1016/j.redox.2019.101126)
Supplement: Supplementary file 1 — Supplementary material [file mmc1.pdf]

High glutathionylation of placental endothelial nitric oxide synthase in preeclampsia

Paul Guerby, Audrey Swiader, Nathalie Augé, Olivier Parant, Christophe Vayssière, Koji Uchida, Robert Salvayre, Anne Negre-Salvayre

SUPPLEMENTAL RESULTS

Supplemental Figure I

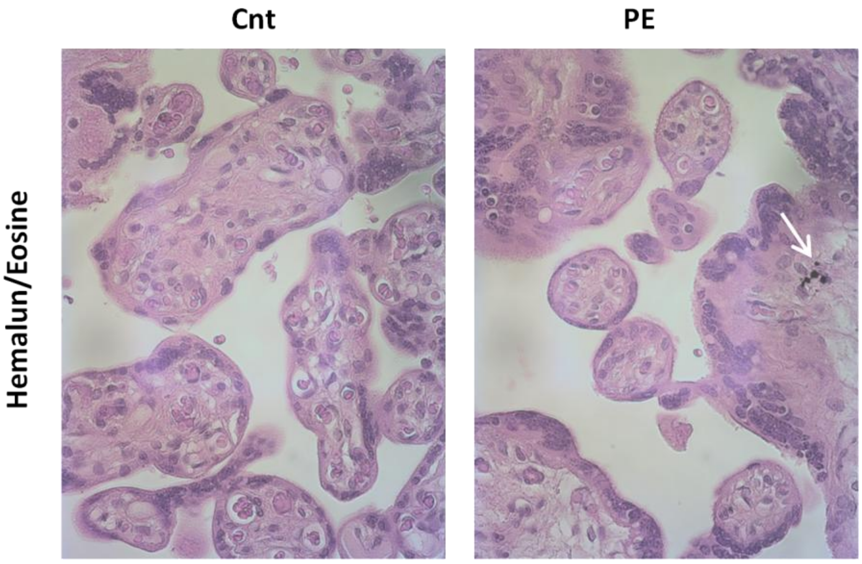

Supplemental Figure I: Immunohistological features of control and PE placentas

Hemalun/eosin histological staining in control and PE placentas. The arrow indicates the presence of calcification clusters, characteristic of PE.
These pictures are an exemple of 4 separate experiments on control and PE placentas.

Supplemental Figure II

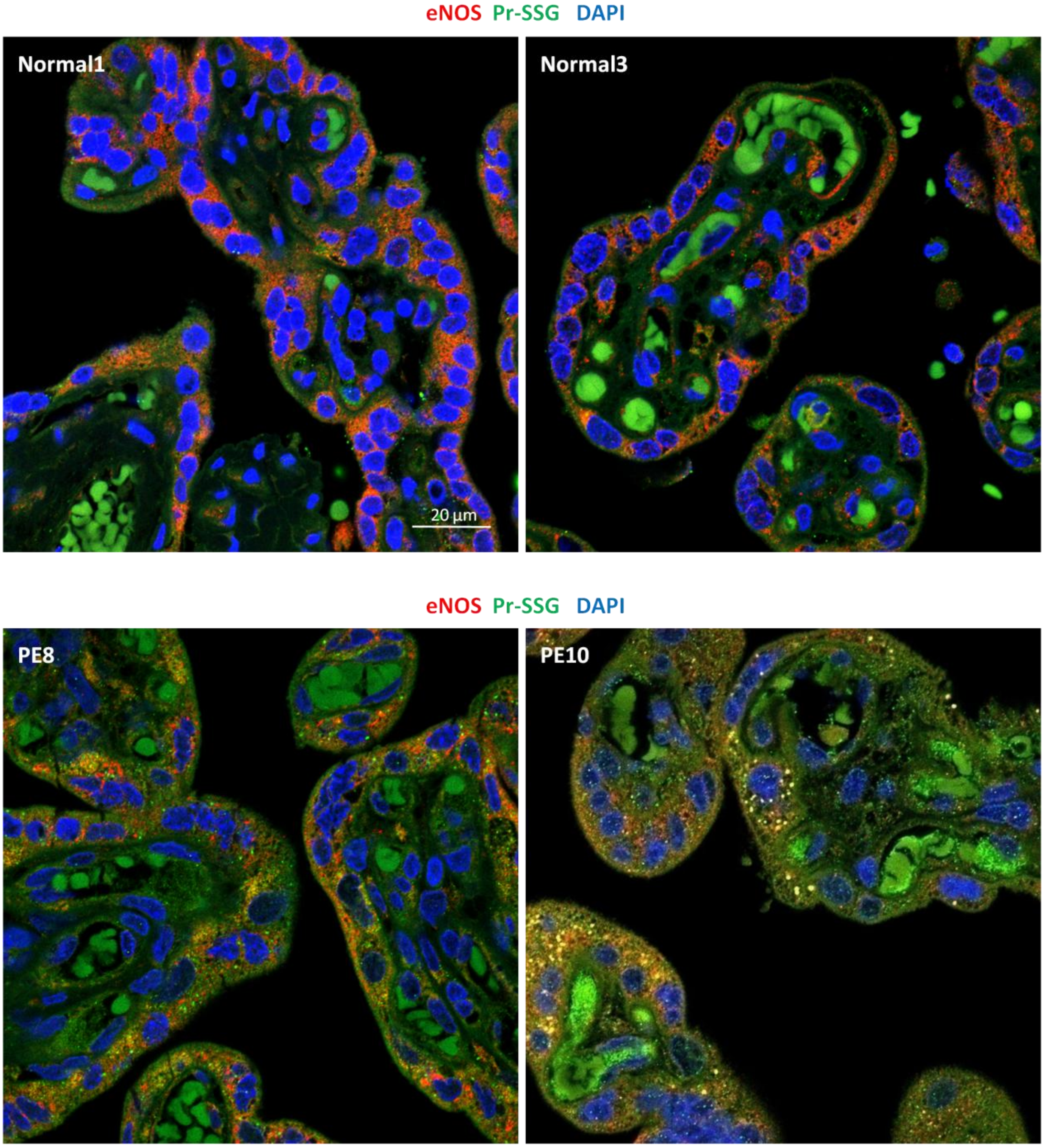

Supplemental Figure II: Representative pictures of eNOS glutathionylation in PE placentas

Supplemental pictures showing the colocalization of eNOS (red) with glutathione (green,) in two different normal (upper panels) and two different PE placentas (lower panel), treated as described in the legend to Figure 1.

Supplemental Figure III: Representative pictures of eNOS and vWF staining in PE placentas

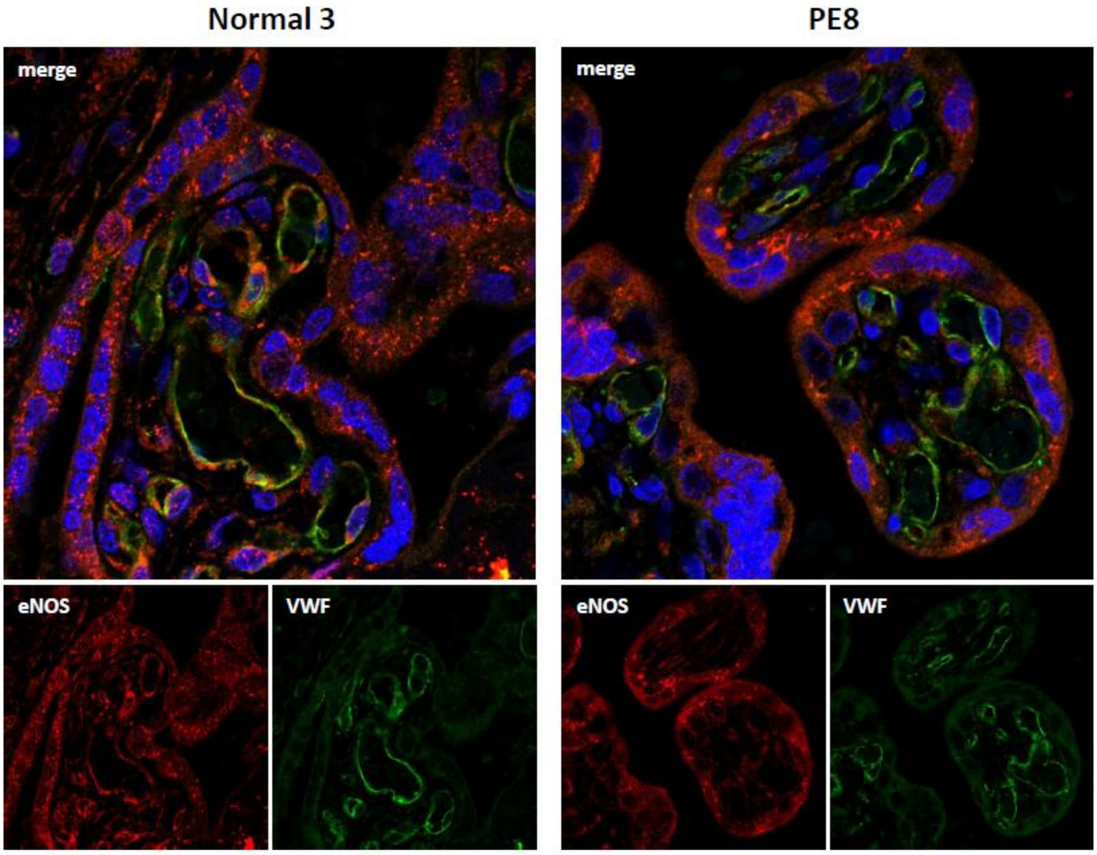

Supplemental Figure III: Colocalization of eNOS and the Von Willebrand Factor

Immunofluorescence and confocal microscopy pictures showing the expression of eNOS (red) and von Willebrand factor (VWF), and the merge in normal (left panels) and PE placentas (right panels), treated as described in the legend to Figure 1.
These pictures are representative of the analysis of 3 normal and 3 PE placentas

Supplemental Figure IV

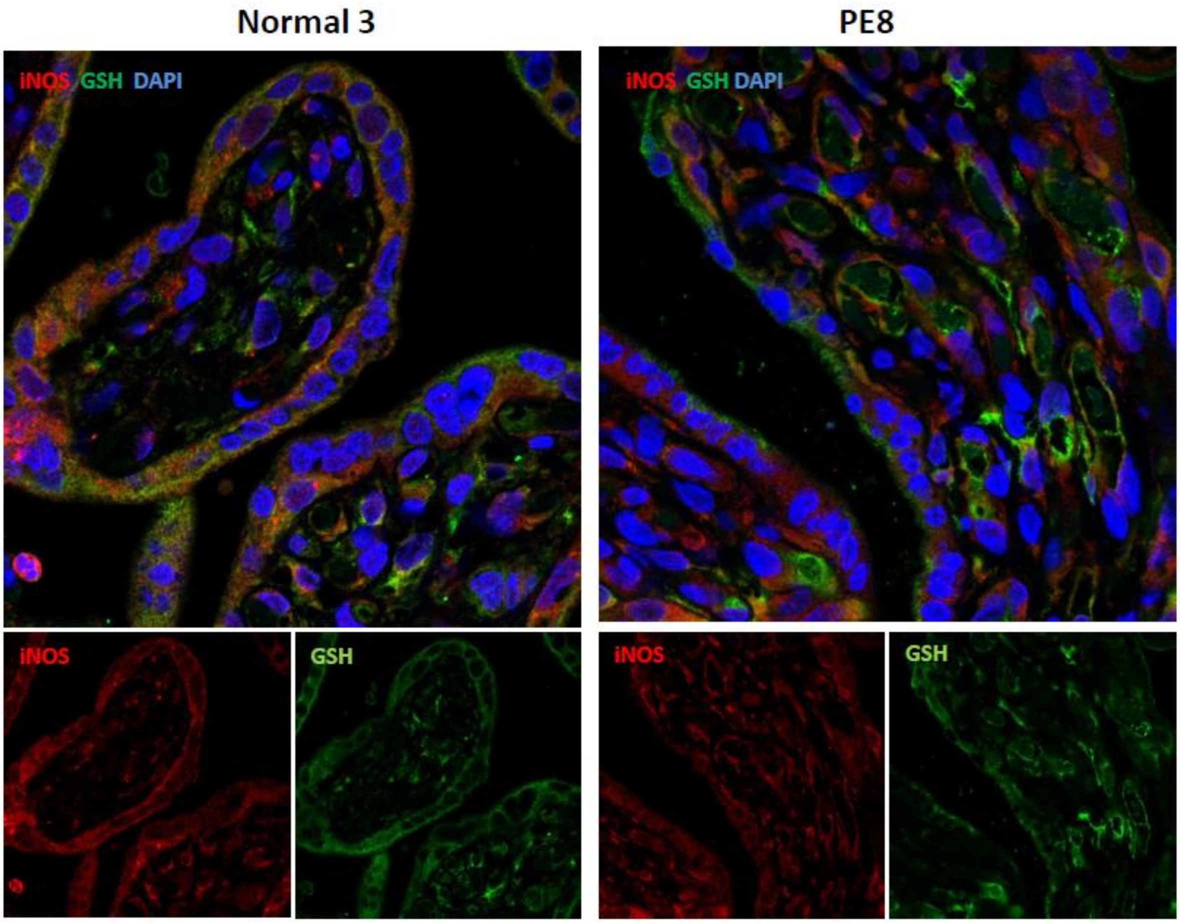

Supplemental Figure IV: iNOS expression and glutathionylation in placentas

Immunofluorescence and confocal microscopy pictures showing the expression of iNOS (red) and GSH, and the merge in normal (left panels) and PE placentas (right panels), treated as described in the legend to Figure 1.

These pictures are representative of the analysis of 3 normal and 3 PE placentas

Supplemental Figure V

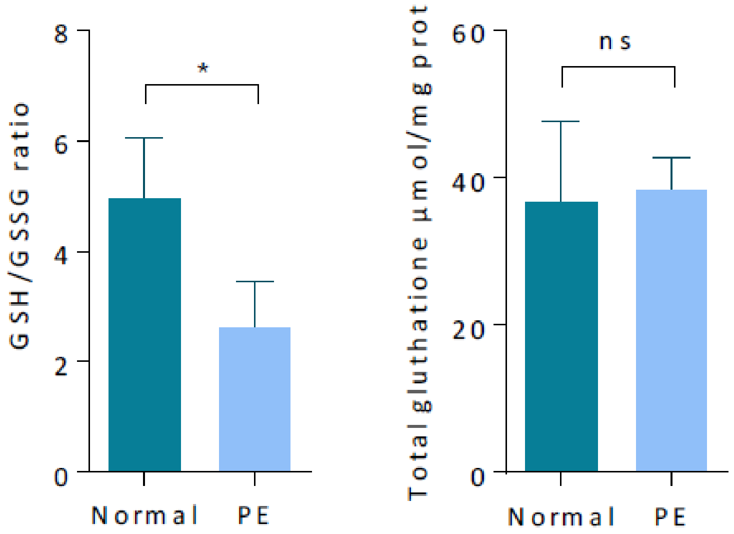

Supplemental Figure V. Total glutathione content in placentas
Reduced/oxidized glutathione ratio, and total glutathione (detected using the GSH Detection Assay Kit Abcam),were measured in normal (n=4) and PE (n=4) placenta homogenates. Data are expressed as means ± SEM, and statistical analysis was assessed using a Mann-Whitney test. *$p < 0.05$; ns, non significant.

Supplemental Figure VI

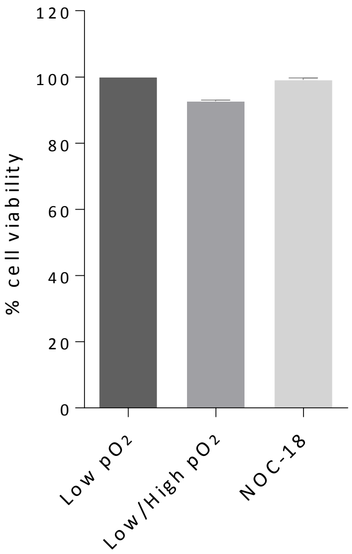

Supplemental Figure VI: Viability of HTR8 exposed to low pO_2 and pO_2 changes and NOC-18

The viability of HTR8 exposed for 48h to low pO_2 and pO_2 change conditions and NOC-18 (5µM, $O_2$1%), was evaluated using the MTT [3-(4,5 dimethylthiazol-2-yl)-2,5-diphenyl tetrazolium bromide] test. The results are expressed as % of the low pO_2 control, means ± SEM of 4 separate experiments.

Supplemental Figure VII

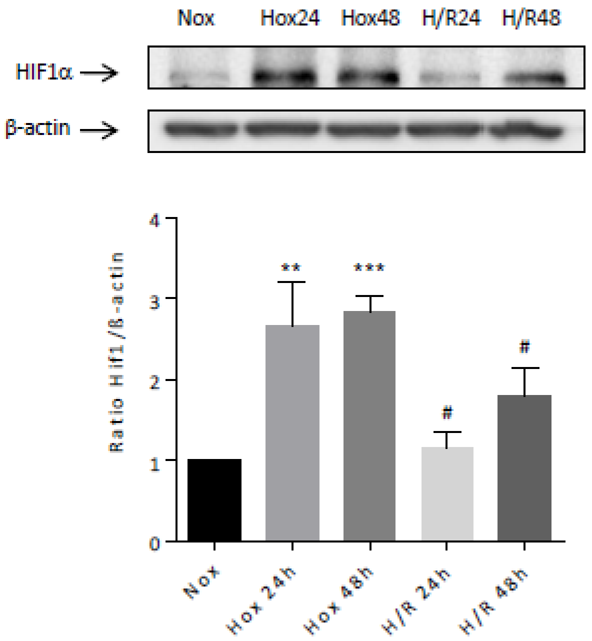

Supplemental Figure VII: Expression of HIF1α in HTR8 exposed to low pO_2 and pO_2 changes

The expression of HIF1α was investigated by western-blot of cell homogenates from HTR8 exposed for 24h or 48h to high pO_2 (20%, Nox), low pO_2 (1%, Hox) and pO_2 change conditions (H/R). The results are representative of 4 separate experiments. Means ± SEM of 4 separate experiments, statistical analysis with a Student t-test. $^{\#}$, $p<0.05$; **, $p<0.01$; ***, $p < 0.001$.

Supplemental Figure VIII

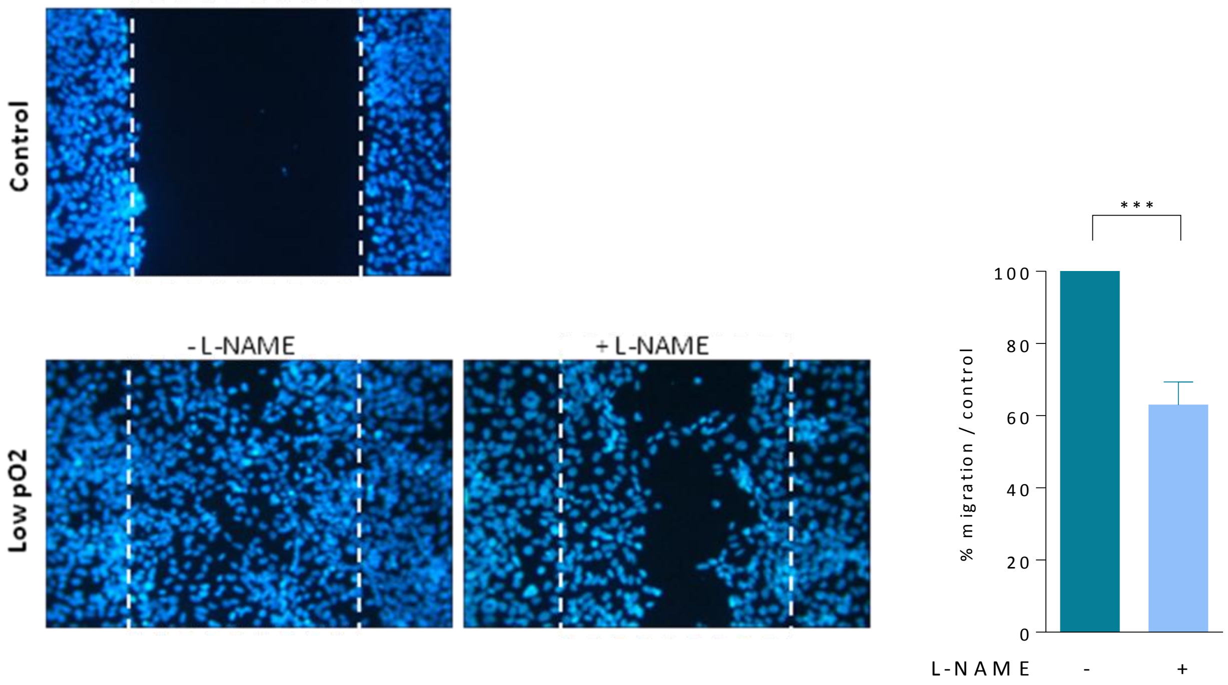

Supplemental Figure VIII: Effect of L-NAME on HTR8 migration in the wound closure assay

The wound-closure assay was performed as described in the Methods Section and in Fig.5, and HTR8 were maintained in low pO_2 for 48h in the presence of L-NAME (100 µM). The quantification of HTR8 migration was done by counting the cells having migrated within the wound. Data are means ± SEM and representative images of 4 separate experiments. Statistical analysis with a Student t-test, ***p<0.001.
